# Successful Aging: Re-Conceptualized

**DOI:** 10.1093/geroni/igaa057.2258

**Published:** 2020-12-16

**Authors:** Alexander Eustice-Corwin, Rachel Missell, Silvia Sörensen

**Affiliations:** University of Rochester, Rochester, New York, United States

## Abstract

A review of the aging literature yields 105 operational definitions of “successful aging” in use. These theoretical discrepancies have caused some investigators to question the utility of the concept (Cosco et al., 2014). Investigators who remain committed to the concept acknowledge its conceptual messiness, but have found no consensus for resolution. We propose a revised concept of successful aging, combining a life-course perspective (Rowe & Kahn, 2015) with a neo-Aristotelian theoretical framework. Such a framework justifies certain changes to the definition of “successful aging;” it situates our concept of successful aging within a broader view of human development, is more inclusive, and suggests empirically adequate research questions. Specifically, conceptualizing “successful aging” along neo-Aristotelian lines means defining it as the maintenance of proper human functioning across the life-course and into late adulthood. For Aristotle, proper human functioning entails realizing one’s potential as a “rational social animal,” with rational implying goal-oriented thinking, means-ends reasoning, other forms of instrumental rationality (not excluding emotionality). Social suggests active engagement in a community, within the limits of an individuals’ comfort and ability. These two criteria determine “success” in older age. Recent research on successful aging reveals that absence of disease and disability does not appear to be a constituent of “successful aging.” Therefore, physical health is neither necessary nor sufficient for “success.” Our re-conceptualization of “successful aging” could be tested using confirmatory factor analysis, with social and reasoning/problem-solving factors loading onto a second order Successful Aging factor. This understanding allows for greater empowerment of older adults.

